# Assessment of Microbiome-Based Pathogen Detection Using Illumina Short-Read and Nanopore Long-Read Sequencing in 144 Patients Undergoing Bronchoalveolar Lavage in a University Hospital in Germany

**DOI:** 10.3390/ijms26209841

**Published:** 2025-10-10

**Authors:** Merle Bitter, Markus Weigel, Jan Philipp Mengel, Benjamin Ott, Anita C. Windhorst, Khodr Tello, Can Imirzalioglu, Torsten Hain

**Affiliations:** 1 Institute of Medical Microbiology, Medical Microbiome-Metagenome Unit (M3U), Justus Liebig University Giessen, 35392 Giessen, Germany; merle.bitter@posteo.de (M.B.); markus.weigel@mikrobio.med.uni-giessen.de (M.W.); janphilipp.mengel@hlfgp.hessen.de (J.P.M.); benjamin.ott@mikrobio.med.uni-giessen.de (B.O.); 2 German Center for Infection Research (DZIF), Partner Site Giessen-Marburg-Langen, Justus Liebig University Giessen, 35392 Giessen, Germany; 3 Institute of Medical Informatics, Justus Liebig University Giessen, 35392 Giessen, Germany; anita.c.windhorst@informatik.med.uni-giessen.de; 4 Department of Internal Medicine, Universities of Giessen and Marburg Lung Center (UGMLC), German Center for Lung Research (DZL), Justus Liebig University Giessen, 35392 Giessen, Germany; khodr.tello@innere.med.uni-giessen.de

**Keywords:** lower respiratory tract infection, bronchoalveolar lavage fluid, microbiome, 16S rRNA gene, Illumina short-read sequencing, nanopore long-read sequencing, culture-based diagnostics

## Abstract

Lower respiratory tract infections (LRTIs) represent a significant global health concern, and the accurate identification of pathogens is crucial for patient care. Culture-based methods are the gold standard, but their detection abilities are limited. Next-generation sequencing (NGS) offers a promising method for comprehensive microbial detection, providing valuable information for clinical practice. In this study, 144 bronchoalveolar lavage fluid samples were collected, culture-based diagnostics were performed, and bacterial microbiome profiles were generated by short-read sequencing of the V4 region of the 16S rRNA gene using Illumina technologies and long-read sequencing with Oxford Nanopore Technologies (ONT) to determine the full-length 16S rRNA gene. The most common genera detected by NGS included Streptococcus, *Staphylococcus*, *Veillonella*, *Prevotella*, *Rothia*, *Enterococcus*, and *Haemophilus*. Short-read sequencing detected cultured bacteria at the genus level in ~85% of cases, while long-read sequencing demonstrated agreement with cultured species in ~62% of cases. In three cases, long-read sequencing identified the uncommon potential lung pathogen *Tropheryma whipplei* not detected with traditional culturing techniques. The NGS results showed a partial overlap with culture as the current diagnostic gold standard in LRTI. Additionally, NGS detected a broader spectrum of bacteria, revealed fastidious potential pathogens, and offered deeper insights into the complex microbial ecosystem of the lungs.

## 1. Introduction

The lung microbiome is one of the least populated microbiomes in the human body [1,2,3] and has not been explored as thoroughly as other bodily habitats such as the gut [4,5,6]. However, accumulating knowledge over the last decade has highlighted the (patho-)physiological importance of the lung microbiome in health and in acute and chronic disease [2,7]. The microbiome of the healthy lower respiratory tract is very dynamic and transient, with high bacterial diversity and low bacterial density [2,4,8]. In pneumonia, the local microbiome structure in the lung is scrambled, allowing one or a few pathogenic taxa to predominate [9,10]. The conventional linear model, in which a pathogen enters the lung and triggers an infection, has gradually been replaced by a more dynamic, multilayer concept of pathogenesis [10,11]. Moreover, the clear distinction between pathogenic and non-pathogenic organisms is increasingly being blurred, as common pathogens associated with lower respiratory tract infections (LRTIs), such as *Streptococcus pneumoniae*, are frequently present in the airways of asymptomatic individuals [6,10,12].

This paradigm shift has been considerably propelled by the introduction of next-generation sequencing (NGS) [2,9,10]. The combination of massively parallel sequencing techniques and bioinformatic data analysis has fundamentally transformed both understanding and research of the human microbiome [2,8,13]. While previous diagnostic techniques target a specific pathogen a priori, NGS embodies a broader approach by mapping the lung microbiome as a complex community [10].

LRTIs are a leading contributor to morbidity and mortality worldwide. In 2021, the global incidence of non-COVID-19 LRTI was estimated at 344 million episodes, resulting in 2.18 million deaths, primarily among vulnerable populations such as infants, the elderly, and those affected by air pollution or extreme poverty by air pollution or extreme poverty [14,15]. The precise and streamlined identification of causative agents in LRTI is crucial for effective patient care. However, pathogens often go undetected by the existing culture-based methods [16]. In their systematic review, Shoar and Musher demonstrated that in over half of the cases studied, conventional methods such as culturing and PCR failed to find an etiologic microbe for community-acquired pneumonia [17]. In addition, certain bacteria, such as *Bartonella*, *Legionella*, *Mycobacterium,* and other atypical bacteria, are challenging to cultivate [18,19,20].

NGS techniques offer a promising approach for improving pathogen detection in samples acquired from the respiratory tract and other parts of the body, as demonstrated by numerous studies [21,22,23,24,25,26,27,28]. Furthermore, they could provide valuable information for clinical practice, such as microbial diversity or DNA concentration as an indicator of exceeding microbial growth [29,30]. Overall, this could lead to more personalised patient care [31,32,33].

This study aimed to systematically compare the bacterial microbiome profiles of bronchoalveolar lavage fluid (BALF) samples using Illumina short-read sequencing of the V4 region of the 16S rRNA gene, long-read sequencing of the full-length 16S rRNA gene via Oxford Nanopore Technologies (ONT) and culture-based routine diagnostics, employing a rapid ONT sequencing workflow for pathogen detection.

Moreover, we assessed whether DNA concentration and α-diversity varied across samples that grew a potential pathogen, a commensal, or had no growth in the culture.

## 2. Results

**Figure 1:**
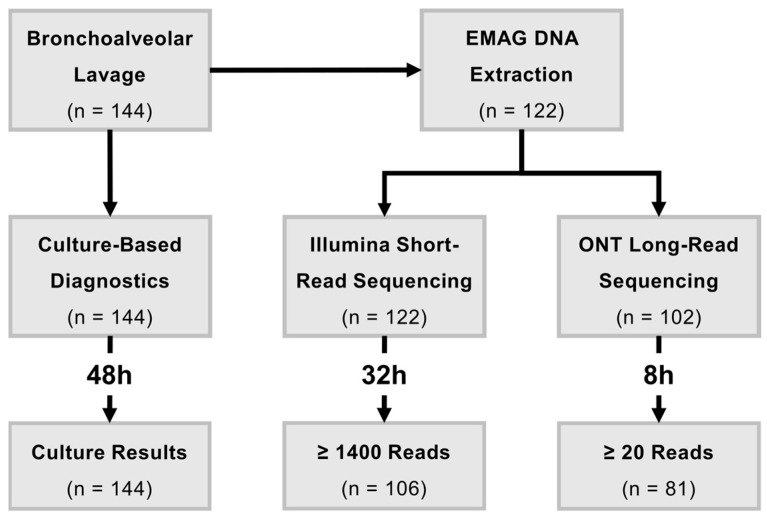
A total of 144 BALF samples were collected and have undergone routine diagnostics. Of those, 122 samples were used for Illumina short-read sequencing and 102 for ONT long-read sequencing, resulting in 106 and 81 samples being included in this study, respectively.

From December 2020 to October 2021, 144 BALF samples from patients undergoing bronchoalveolar lavage at the University Hospital Giessen (Germany) were collected. These samples were cultured and reported within 24 to 48 h by the Institute of Medical Microbiology’s routine diagnostic unit (Supplementary Figure S1a). A total of 22 samples had to be excluded from this study due to there being an insufficient amount of fluid (12) or a positive test for SARS-CoV-2 (10). The remaining 122 samples were sequenced with Illumina short-read sequencing, resulting in 106 samples with at least 1400 sequence reads included in the analysis. The Illumina workflow for sample processing, sequencing, and analysis took approximately 32 h (Supplementary Figure S1b). As a final part of the study, 102 of the 122 included patient samples were sequenced with ONT long-read sequencing. A total of 20 samples had to be excluded due to insufficient remaining DNA extracts for further sequencing. In total, 82 BALF samples reached the minimum threshold of 20 classified sequence reads and were therefore included in the analysis. The microbiome profiles for these samples were generated within 8 h, which limited sequencing to two hours. A total of 82 BALF samples reached the minimum threshold of 20 classified sequence reads and were therefore included in the analysis. The microbiome profile for these samples was within 8 h, which limited sequencing to two hours (Supplementary Figure S1c). In this study, we focused on the 106 samples for which we obtained overlapping NGS and culture results (Figure 1).

### 2.1. Culture-Based Diagnostics

Bacterial growth was observed in 67 samples (63.2%), with a total of 28 species identified in culture. The most frequently reported taxa included α-haemolytic *Streptococcus*, saprophytic *Neisseria*, and *Staphylococcus aureus*. Bacteria detected by culture were assessed for pathogenicity according to the S3 guidelines for community- and hospital-acquired pneumonia, using additional sources when guideline information on specific species was unavailable (Supplementary Table S1), resulting in 9 of the 28 cultured species being classified as potentially pathogenic. In 26 samples (24.5%), at least one potential pathogen could be identified (Supplementary Table S2).

### 2.2. Results of Illumina 16S rRNA Gene V4 Region Sequencing

**Figure 2:**
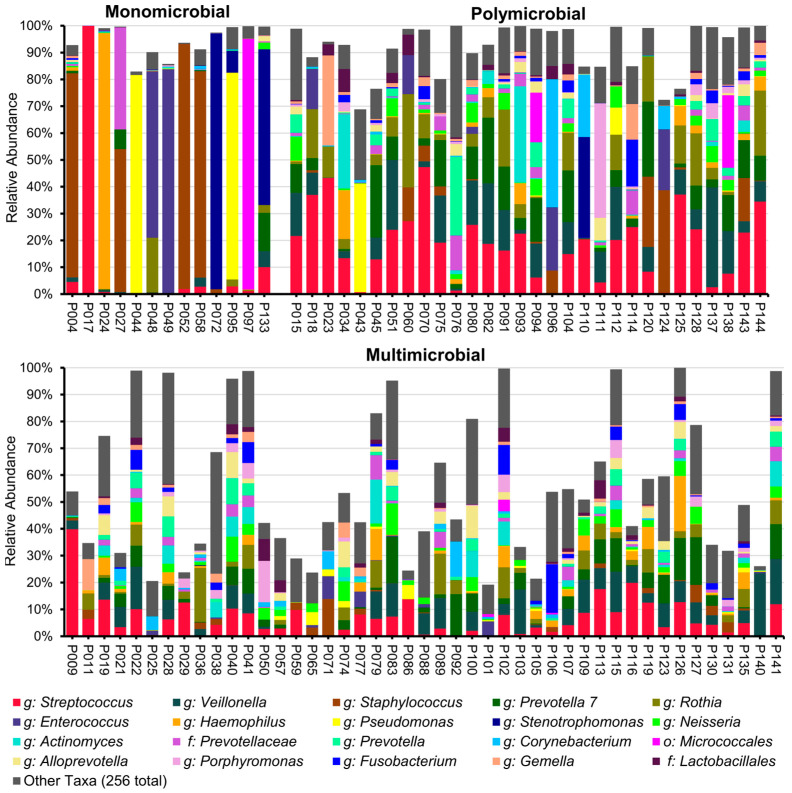
Taxonomic bar charts of Illumina short-read sequencing of the V4 region of the 16S rRNA gene after the removal of detected contaminants. The missing parts of the bars indicate the proportion of contamination removed.

The 16S rRNA gene sequencing of the V4 region using Illumina short-read sequencing technology yielded a total of 685,459 sequence reads, with a median of 5314 sequence reads per sample (IQR, 3344–8471). The genera most frequently identified through Illumina sequencing included *Streptococcus*, *Staphylococcus*, *Veillonella*, *Prevotella* 7, *Rothia*, and *Enterococcus*. We categorised our microbiome profiles generated by Illumina short-read and ONT long-read sequencing into four groups (see Section 4.5 and Supplementary Table S3). A total of 13 BALF samples displayed a monomicrobial profile, while 30 samples showed a polymicrobial profile. Additionally, 45 BALF samples were characterised by a multi-microbial profile, and 18 samples were included in the low-biomass group (Supplementary Figure S2). The monomicrobial samples demonstrated the presence of the genera *Staphylococcus* (4), *Pseudomonas* (2), *Stenotrophomonas* (2), *Enterococcus* (2), *Haemophilus* (1), and *Streptococcus* (1), as well as bacteria of the order *Micrococalles* (1) (Figure 2, Supplementary Table S2).

### 2.3. Results of ONT 16S rRNA Gene Full-Length Sequencing

**Figure 3:**
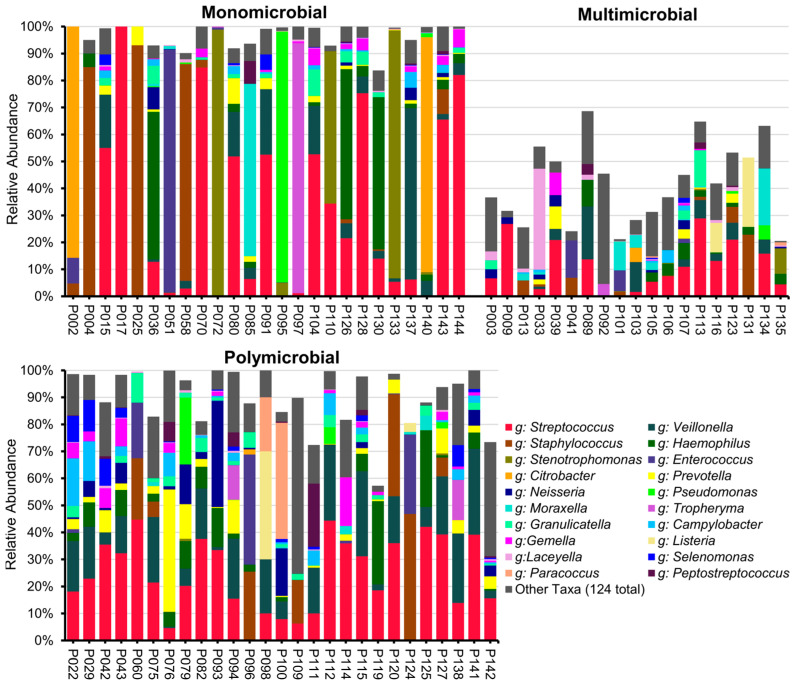
Taxonomic bar charts of ONT long-read sequencing of the full-length 16S rRNA gene after the removal of detected contaminants limited to genus resolution. The missing parts of the bars indicate the proportion of contamination removed.

The ONT long-read sequencing of 102 samples produced 81 samples with a minimum sequence read count of 20, which were included in this analysis. For those 81 samples, a total of 398,144 sequence reads were generated, with a median of 329 sequence reads per sample (IQR, 109–1279). A total of 322 distinct species were observed, representing 144 genera. The three most abundant genera were *Streptococcus*, *Veillonella,* and *Staphylococcus*, which is in line with the Illumina results. In contrast to the culture results, Neisseria was not among the most abundant genera in the sequencing results. On the genus level, 25 samples were categorised as monomicrobial, 27 as polymicrobial, 19 samples showed a multi-microbial profile, and 10 samples were of the low-biomass category (Figure 3, Supplementary Figure S3). While most of the detected species were commensals of the upper respiratory tract, we detected one or more potential pathogens in 22 BALF samples (27.2% of the included BALF samples). Three potential pathogens identified using ONT long-read sequencing were not observed in culture, namely, *Listeria monocytogenes*, *Streptococcus pseudopneumoniae*, and *Tropheryma whipplei* (Supplementary Table S2).

### 2.4. DNA Yield of BALF Samples

**Figure 4:**
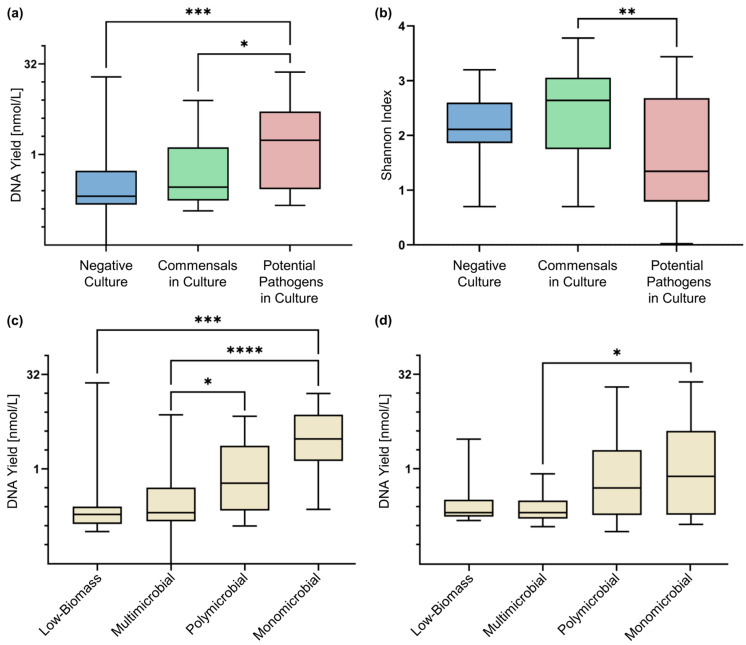
Overview of DNA concentration (**a**) and Shannon diversity index for Illumina short-read sequencing results (**b**) from extracted BALF samples, which are sorted by culture result and plotted with their medians. DNA concentration of the extracts was sorted by microbiome profile category for Illumina short-read sequencing (**c**) and ONT long-read sequencing (**d**). DNA concentration is plotted on a logarithmic scale (base two). Asterisks indicate statistically significant differences. * = *p* ≤ 0.05; ** = *p* ≤ 0.01; *** = *p* ≤ 0.001; **** = *p* ≤ 0.0001.

We categorised our microbiome profiles generated by Illumina short-read and ONT long-read sequencing into four groups (see Section 4.5 and Supplementary Table S3). The DNA yield in samples in which a potential pathogen was cultivated was significantly higher compared to samples with either negative culture results or those containing commensal bacteria (Figure 4a). In samples sequenced by short-read sequencing, the Shannon index indicated a lower diversity in samples containing potential pathogens in culture compared to samples with commensals (Figure 4b), suggesting the presence of a few dominant bacteria.

The DNA yield of the monomicrobial classified samples was the highest in short-read and long-read sequencing, followed by the polymicrobial samples, indicating that the DNA yield is the highest in samples where a small number of bacterial species are dominant. The low-biomass samples, along with the multi-microbial samples, produced the lowest DNA yield, which aligns with the observation that contaminations were predominant in these types of samples (Figure 4c,d).

### 2.5. Validation of Culture-Based Diagnostics with NGS

To investigate to what extent culture results match NGS data, Table 1 was established. It shows the species detected in the culture alongside their matching species via ONT sequencing and their genera via Illumina short-read sequencing. This study generated one to four culture results for 67 of the 106 BALF samples, leading to a total of 118 positive culture reports. Because of the lower number of ONT-sequenced samples included in this study, long-read sequencing covers only 50 culture-positive samples with 91 positive culture reports.

A corresponding genus was identified in 100 out of 118 cases (84.7%) by short-read sequencing. In 51 cases (43.2%), these genera showed a relative abundance of 10% or more. All 35 samples with a culture report of α-haemolytic *Streptococcus* had a corresponding genus in the Illumina short-read sequencing data, albeit at varying ranks. For *S. aureus* and coagulase-negative *Staphylococcus*, the corresponding genus was missing in 35% of cases. Among the 27 reports of potential pathogens, 9 lacked a corresponding genus in short-read sequencing (33.3%). Neither of the two *Citrobacter koseri* reports, nor the *Serratia marcescens* report from routine diagnostics, could be validated with Illumina 16S rRNA gene sequencing data. Furthermore, two reports for *Escherichia coli* could not be confirmed because of the presence of this species in the negative controls (Table 1, Supplementary Table S2).

A total of 56 of the 91 culture-positive reports corresponding to the long-read-sequenced samples matched on species level (61.5%); however, 8 of these reports were verified with fewer than 10 sequence reads. For α-haemolytic *Streptococcus*, 24 of 26 reports were covered by ONT long-read sequencing (92.2%). Coagulase-negative *Staphylococci* were not detected by long-read sequencing in two cases. The *S. aureus* determination was limited to three out of eight culture reports (37.5%). In contrast to short-read sequencing, both reports of *C. koseri* were also confirmed by long-read NGS. The detection of *E. coli* was not feasible due to the overlap with the negative controls. The cultured species Neisseria flava could not be retraced by Nanopore sequencing; however, *Neisseria flavescens* and *Neisseria perflava* were sequenced in these two samples instead. In a similar fashion, the third *Stenotrophomonas maltophilia* was detected as *Stenotrophomonas* sp. MYb57 (Table 1, Supplementary Table S2).

### 2.6. Expanding Bacterial Pathogen Detection by NGS

The limited resolution of Illumina short-read sequencing of the V4 region of the 16S rRNA gene makes the differentiation between pathogens and commensals of the same species impossible. in many cases. The uncommon potential pathogen *T. whipplei*, not found by routine culture diagnostics, was an exception to this and was detected by short-read NGS with an extended classification using BLASTn [34].

**Figure 5:**
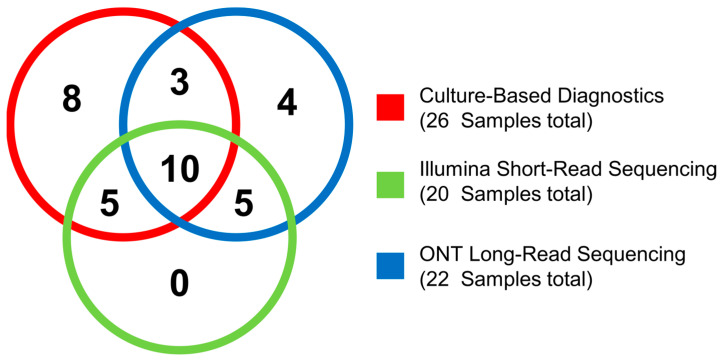
Venn diagram showing the overlap of samples containing a potential pathogen between culture-based diagnostic and ONT long-read sequencing on species level. Illumina short-read sequencing has been assessed on genus level.

ONT long-read 16S rRNA gene full-length sequencing uncovered potential pathogens in nine BALF samples not detected by culture. In seven of those nine cases, we found a potential pathogenic species that was not detected in any culture result (*L. monocytogenes*, *Prevotella oris*, *Streptococcus pseudopneumoniae*, *T. whipplei*, and *Streptococcus agalactiae*). Four of those nine culture-negative cases could not be confirmed by Illumina short-read sequencing on genus level. These were *Haemophilus influenzae* (1), *L. monocytogenes* (2), and *S. aureus* (1) (Figure 5). In three cases, the potential pathogens detected in the culture and via long-read sequencing did not match (Supplementary Table S2).

## 3. Discussion

NGS has become a key driver of advancements in microbiological research and holds significant promise for pathogen detection in the future. In this study, we showed that the most frequently detected genera by targeted 16S rRNA gene sequencing from patient BALF samples were *Streptococcus*, *Staphylococcus*, and *Veillonella*, which aligns with findings from several previous studies [8,35,36].

A total of 31 of the 82 BALF samples sequenced with Illumina and ONT agreed on the most abundant genera, which could be due to the different 16S rRNA gene primers used in the two sequencing approaches. Additional factors, such as genomic DNA extraction, the library construction, the sequencing platform, and the bioinformatic processing workflow, can influence the sequencing results and pathogen detection [31,37,38,39,40]. When aiming for a future routine application of NGS, this has to be taken into account, and standardised protocols have to be established. A combination of different NGS techniques or NGS and culture-based diagnostics can harden a diagnostic strategy against single points of failure, such as incomplete primer coverage, low sequencing depth, or unculturable bacteria.

To systematically compare culture results with Illumina and ONT sequencing, Table 1 was generated, which illustrates the overlap between the species identified in culture and the taxa detected by NGS. The detection rate of NGS (Illumina: 84,7%; ONT: 61,5%) was lower than in similar studies based on Illumina, such as Zachariah et al. [28], and Yoo et al. [27], or studies based on ONT, such as Lao et al. [41]. False negatives-negative NGS results may be attributed to factors such as low microbial load [27], incomplete primer coverage, and limited sequencing depth. However, false-positive culture results may also be possible.

Four samples revealed a different pathogen in ONT long-read sequencing than that reported by culture-based diagnostics. Five possible pathogens detected by culture had a relative abundance of under 3% in the microbiome profiles, which could diminish the pathogen detection ability of Illumina and ONT sequencing.

However, in several samples, genera of clinical significance, such as *Haemophilus* and *Staphylococcus*, were identified through Illumina sequencing even though culture results were negative. Notably, genus-level resolution is insufficient to distinguish commensals from pathogens such as *S. aureus* from *S. epidermidis*, highlighting the critical need for species-level identification in a clinical setting. Therefore, ONT 16S rRNA full-length gene sequencing was conducted for higher taxonomic resolution. In nine cases, ONT sequencing detected the following potential pathogens that were absent in the culture: *H. influenzae* (1), *L. monocytogenes* (2), *P. oris* (1), *S. aureus* (1), *S. pseudopneumoniae* (1), *T. whipplei* (2), and a coinfection with *S. pseudopneumoniae* and *S. agalactiae* (1). *T. whipplei* was also confirmed by Illumina sequencing. The causative agent of Whipple’s disease typically affects the gastrointestinal tract. Still, colonisation or infection of the lung were observed in a limited number of cases [42]. *L. monocytogenes* cases were only detected by ONT long-read sequencing. This bacterium can cause severe and life-threatening diseases, which generally manifest as bacteraemia and/or meningitis in neonates and elderly and immunocompromised patients; however, pulmonary listeriosis in adults has also been described [43].

The microbiome profiles offered further qualitative and quantitative insights into the bacterial composition, also labelled as “culture-independent indices of infection” [25]. In this study, the DNA yield, reflecting the DNA concentration in BALF extracts, was significantly higher in samples with a potential pathogen in culture (Figure 4a). This observation is consistent with a study by Dickson et al. [25]. However, individual values could not reliably be used as a single discriminator to determine whether a potential infection might be present. Concerning diversity indices, several studies have linked lower diversity indices to pathogen isolation in culture [23,44,45], which could only be partially reproduced by our study (Figure 4b).

The division into multi-microbial, based on the frequency distribution of the bacteria, aimed to place the NGS results in a broader context and to attain more generalisable findings. In addition, it was used for a structured comparison with the culture results. We indicate these profiling parameters in Figure 4c. However, the classification is somewhat artificial, as it oversimplifies the lung microbiome’s complex composition. The division into categories (mono-/poly-/multi-microbial and low-biomass) based on the frequency distribution of the bacteria aimed to place the NGS results in a broader context and to attain more generalisable findings. In addition, it was used for a structured comparison with the culture results. We assumed that the monomicrobial profiles, dominated by one single taxon, indicate a disruption in the microbial balance and therefore severe dysbiosis. Figure 4c,d shows that monomicrobial samples exhibited higher DNA concentrations than the multi-microbial or low-biomass ones, suggesting that these parameters could further aid in evaluating the condition of the lung microbiome. However, the classification is somewhat artificial as it oversimplifies the lung microbiome’s complex composition.

Culture-based standard diagnostics delivered results within 48 h compared to Illumina sequencing with 32 h. The ONT approach provided accurate results within one clinical working day by accepting a reduced sequence read depth in this study, which facilitates a rapid diagnostic turnaround time and enables early therapeutic intervention.

While 16S rRNA gene amplicon sequencing can identify potential pathogens, culture-based diagnosis also facilitates a deeper phenotypical classification, including the detection of antibiotic resistance and the potential virulence of culturable bacteria.

Overall, our findings align with those of previous research, reinforcing that NGS cannot currently completely replace classical bacterial culture techniques as the diagnostic gold standard for microbial detection in LRTI as relevant discrepancies between culture-based methods and NGS remain. However, NGS techniques provide a wealth of data. With increasing knowledge, the improvement of workflows, and decreasing costs, the establishment of NGS gradually comes within reach [22,39,46]. It could prove to be a valuable addition, improving the limited bacterial detection rate of culture, especially with respect to the methods reaching species-level resolution, such as ONT sequencing [27,47,48]. Additionally, NGS can come into play in complex cases, such as in patients pretreated with antibiotics [23,27]. With its fast turnaround time, ONT could play an important role as a point-of-care instrument. Additionally, features in the microbiome (culture-independent indices of infection) could serve as diagnostic or prognostic biomarkers to guide concrete clinical decisions [29,49].

Microbiome studies, including this investigation, have inherent strengths and limitations that influence their findings and interpretations. Our study benefited from a large patient cohort. We did not include a healthy control group; however, the primary objective of this study was to compare the different methods rather than to investigate specific microbiome characteristics associated with disease. A common challenge of 16S rRNA gene sequencing, especially in lower respiratory tract samples, is the high risk of contamination during laboratory procedures [2,9,31,50], which was also observed in our study. To address this, we included serval isolation, no-template, saline solution, and bronchoscope rinse controls. This study employed an observational, cross-sectional design, a common approach in respiratory microbiome research [8,31]; however, longitudinal studies are needed to address questions about causation or the principal (patho-)mechanisms [2,8,31,51]. Additionally, geographical, environmental, and ethnic variations should be investigated further, as current knowledge relies on research populations primarily from more highly developed countries, introducing potential biases [13,52,53].

## 4. Materials and Methods

### 4.1. Sample Acquisition and Culture-Based Diagnostics

Bronchoalveolar lavage was carried out in 144 patients as part of the clinical patient care at the University Hospital Giessen and Marburg, Giessen (UKGM Giessen, Germany). A total of 50 µL BALF samples were inoculated onto six distinct agar plates (MacConkey agar No. 3; Columbia agar with sheep blood PLUS; chocolate agar with Vitox; Schaedler anaerobe KV selective agar with lysed horse blood; Schaedler anaerobe agar with sheep blood, haemin, and vitamin K1; and Sabouraud–gentamicin–chloramphenicol 2 agar) each, incubated as recommended by the manufacturer (Thermo Fisher Scientific, Waltham, MA, USA; bioMérieux SA, Marcy-l’Étoile, France). Species identification was performed after 24 to 48 h using MALDI-TOF (MS Prime, bioMérieux SA, Marcy-l’Étoile, France), following quality-controlled standard diagnostic protocols at the Institute of Medical Microbiology diagnostics department. Subsequently, the remaining sample material was stored at −80 °C for NGS. The cultured bacteria of 106 BALF samples were assessed for potential pathogens (Supplementary Table S1).

### 4.2. DNA Extraction

DNA extraction for 122 BALF samples was performed using the automated extraction platform for nucleic acid purification EMAG (bioMérieux SA, Marcy-l’Étoile, France) following the manufacturer’s instructions. In brief, 250 µL of each sample were combined with Proteinase K to lyse cellular material, viral particles, bacteria, and fungi. Nucleases were inactivated using lysis buffer. Nucleic acids were isolated and purified via magnetic silica particles and wash buffers and eluted in elution buffer. Genomic DNA (gDNA) was then quantified using the Quant-iT PicoGreen dsDNA Assay Kit (Invitrogen, Carlsbad, CA, USA) and the Qubit High Sensitivity Kit (Invitrogen, Carlsbad, CA, USA).

### 4.3. Control of Contaminations

To monitor contaminations and to verify laboratory processes, isolation and no-template controls were included for each processed batch of samples. To assess contamination during the lavages, four bronchoscopy rinse controls and four saline solution controls of the flush solution were gathered over four days and sequenced.

Of the controls included in the Illumina short-read samples, all the bronchoscopy rinse controls and saline solutions controls were not further analysed because of the low number of usable reads contained in those samples. The remaining controls contained between 550 and 7824 sequence reads and were employed to determine possible contaminations (Supplementary Figure S4).

ONT long-read sequencing yielded reads in two no-template controls, with 89 and 192 sequence reads, respectively, and an isolation control with 383 sequence reads; otherwise, the controls were below 20 classified sequence reads and were excluded (Supplementary Figure S5).

### 4.4. Short-Read Sequencing with Illumina

Library preparation for Illumina short-read sequencing was executed according to [54]. Pipetting steps were automated with the Hamilton Microlab STAR (HAMILTON Bonaduz AG, Bonaduz, Switzerland). Amplification of the V4-region of the 16S rRNA gene was performed with 10 µL of input material and 30 cycles for the PCR (2 min at 98 °C, 30 cycles with 10 s at 98 °C, 10 s at 55 °C and 30 s at 72 °C, plus a final step for 5 min at 72 °C). A total of 5 µL of each library was further amplified with a custom set of 10 µmol unique dual indices (Integrated DNA Technologies, Coralville, IA, USA) and NEBNext Ultra II Q5 Master Mix (New England Biolabs, Ipswich, MA, USA) according to the manufacturer’s protocol for NGS PCR to facilitate multiplex sequencing.

Both amplicons and libraries were purified with AMPure XP Reagent (Beckman Coulter, Pasadena, CA, USA) and eluted in Ambion nuclease-free water (Life Technologies, Carlsbad, CA, USA). The quantity and quality of the libraries were assessed with the Fragment Analyzer (Agilent Technologies Inc., Santa Clara, CA, USA), qPCR (Applied Biosystems, Waltham, MA, USA), and the 2100 Bioanalyzer (Agilent Technologies Inc., Santa Clara, CA, USA). Libraries were pooled in equimolar ratios and diluted to a final concentration of 4 nM. Paired-end reads were sequenced on the MiSeq system (Illumina, San Diego, CA, USA) using the MiSeq Reagent Nano Kit v2 (500-cycles) (Illumina, San Diego, CA, USA) and 20% of PhiX Control v3 (Illumina, San Diego, CA, USA). The generated reads were image processed, base called, and demultiplexed prior to analysis.

### 4.5. Bioinformatic Analysis of Illumina Short-Read Sequencing Data

Microbiome analysis was conducted using Mothur (version 1.48.3) [55]. Paired-end reads were merged, and primer regions were removed and filtered to retain an amplicon length of 253 nt ± 10 nt. Sequence reads with ambiguous nucleotides were excluded. Joined paired-end sequence reads were aligned to the SILVA ribosomal RNA gene database (version 138.2) [56] and trimmed to include only the hypervariable region V4. Clustering was performed with a 97% similarity threshold. After chimaeras were removed using VSEARCH (version 2.17.1) [57], operational taxonomic units (OTUs) were determined and classified using the SILVA ribosomal RNA gene database. For downstream analysis, all samples were subsampled to 1.400 sequence reads, and 16 samples with less than 1400 sequence reads were excluded. Mothur was used to calculate α-diversity indices. The representative sequence for each OTU was further analysed by BLASTn (version 2.12.0) [34] against the 16S ribosomal RNA database from the NCBI RefSeq Targeted Loci Project [58]. A total of 32 OTUs that overlapped with the negative controls were flagged as contaminations and removed from further analysis (Supplementary Table S4). Finally, samples were classified as monomicrobial (a single genus above 50%), polymicrobial (maximal three genera representing 50%), and multi-microbial (any other composition above 20%). Samples with less than 20% of the sequence reads remaining after contamination removal were categorised as low-biomass samples.

### 4.6. Long-Read Sequencing with Oxford Nanopore Technologies

To amplify the entire 16S rRNA gene from the remaining extracted gDNA of the 102 BALF samples, the 16S Barcoding Kit 24 V14 was used according to the manufacturer’s protocol (Oxford Nanopore Technologies, Oxford, UK). For samples with a DNA yield lower than recommended, 15 µL were used as input for the PCR. The libraries before and the after pool normalisation in equimolar ratios were quantified with the Qubit High Sensitivity Kit (Invitrogen, Carlsbad, CA, USA). Samples were sequenced on the MinION Mk1B (Oxford Nanopore Technologies, Oxford, UK) with corresponding MinION Flow Cells (Oxford Nanopore Technologies, Oxford, UK). Between sequencing steps, the flow cell was washed and stored according to instructions for the Flow Cell Wash Kit (Oxford Nanopore Technologies, Oxford, UK). All reagents and flow cells used were compatible with the current chemistry type R10.4.1.

### 4.7. Bioinformatic Analysis of ONT Long-Read Sequencing Data

To assess the quality of long-read sequencing data, fastplong (version 0.3.0) was employed [59]. Sequence reads were filtered for a minimum mean quality of Q20 and a minimum read length of 1000 nucleotides. A total of 21 samples with less than 20 sequence reads remaining after quality control were excluded from this study. Detected adapter and primer sequences were removed. Emu (version 3.5.1) [60] was used to classify the resulting reads to species-level with a combination of the rrnDB [61] and 16S RefSeq records from the targeted loci project [58] as reference database. Reads with the classification *Delftia acidovorans*, *Escherichia coli*, *Paracoccus angustae*, and *Paracoccus marinus* were removed as possible contaminations. Samples were classified as mono-/poly-/multi-microbial and low-biomass, as described for the analyses of Illumina short-read sequencing.

### 4.8. Comparitive Microbiome Analysis

DNA burden and diversity indices were evaluated alongside the culture results. The overlap between the findings from culture-based diagnostics and Illumina short-read sequencing was then assessed in two ways. The first approach involved creating a table to match the species identified in the culture with their respective genera, resembling the analysis conducted by Zachariah et al. The second approach categorised microbiome profiles into four groups (mono-/poly-/multi-microbial and low-biomass) and compared the results to culture outcomes. Subsequently, the microbiome profiles of the full-length 16S rRNA gene of the selected samples were analysed at the species level. Lastly, results from the culture, Illumina short-read, and ONT long-read sequencing were juxtaposed.

### 4.9. Statistic Evaluation

Exploratory data analysis was performed. Statistical significance was set at *p* < 0.05, with a threshold of *p* < 0.1 for preliminary tests (e.g., normality tests). The Kruskal–Wallis test was used to compare three or more independent groups. Dunn’s post hoc test with Bonferroni correction was applied for multiple comparisons. Data were log-transformed (base 2) to stabilise variance. Statistical analyses and figures were generated using GraphPad Prism (version 10.2.3).

## 5. Conclusions

Next-generation sequencing is a promising tool for LRTI diagnostics in clinical microbiology. First, advancing the speed and efficiency of NGS protocols is crucial to facilitate real-time diagnostics, which would enable clinicians to make quicker decisions in acute care settings. Additionally, leveraging NGS to explore the human microbiome could yield insights into disease susceptibility and reveal novel pathogen–host interactions, potentially leading to the identification of previously unknown infectious agents. Furthermore, utilising NGS to monitor and understand antimicrobial resistance—e.g., applying metagenomic approaches—could significantly enhance our ability to track resistance patterns and inform more effective treatment strategies and public health policies. Personalised medicine stands to benefit greatly from NGS by using individual genetic and LRTI microbiome profiles to tailor treatments, offering precision diagnostics that account for personal variability in disease response. Improving accessibility and reducing the cost of NGS technology is also vital, making it feasible for widespread use. Finally, collaborative efforts across disciplines—including genomics, bioinformatics, and clinical medicine—are essential to translate these research advancements into practical and impactful clinical applications.

## Figures and Tables

**Table 1:** Culture-based diagnostic results listed with the respective categories from Illumina short-read sequencing and ONT long-read sequencing. The below-3% category also includes hits with less than ten sequence reads for ONT sequencing. Potential pathogenic bacteria are marked in bold. *E. coli* was excluded because of the overlap with detected contamination in NGS.

Culture-Based Diagnostics		Corresponding Genera Detected by Illumina Short-Read Sequencing					Corresponding Species Detected by ONT Long-Read Sequencing				
Species	No. of Reports	>20%	≥10% <20%	≥3% <10%	<3%	Absent	>20%	≥10% <20%	≥3% <10%	<3%	Absent
***A. odontolyticus***	1	-	-	-	1	-	-	-	-	-	-
***B. bronchiseptica***	1	-	-	1	-	-	-	-	1	-	-
***C. koseri***	2	-	-	-	-	2	2	-	-	-	-
***E. faecalis***	2	1	-	-	-	1	1	-	-	-	1
***E. faecium***	4	2	-	1	1	-	-	1	-	-	2
***E. coli***	2	-	-	-	-	-	-	-	-	-	-
***H. influenzae***	1	1	-	-	-	-	-	-	-	-	-
***H. parahaemolyticus***	1	1	-	-	-	-	1	-	-	-	-
***L. reuteri***	1	-	1	-	-	-	-	-	-	-	-
***L. salivarius***	1	-	-	-	-	1	-	-	-	-	1
**Saproph. *Neisseria***	12	-	-	6	3	3	-	1	1	2	5
***N. flava***	2	1	1	-	-	-	-	-	-	-	2
***P. melaninogenica***	5	-	1	3	1	-	-	-	-	3	1
***P. aeruginosa***	5	3	-	-	1	1	1	-	-	-	3
***Rothia* sp.**	3	1	-	2	-	-	-	-	-	-	2
***R. mucilaginosa***	5	1	3	1	-	-	-	-	-	2	2
***S. marcescens***	1	-	-	-	-	1	-	-	-	-	1
**Coagulase-neg. *Staphylococcus***	9	2	-	1	2	4	2	-	1	3	2
***S. aureus***	11	3	1	-	4	3	1	-	1	1	5
***S. epidermidis***	4	-	1	1	2	-	1	-	-	1	1
***S. maltophilia***	3	3	-	-	-	-	2	-	-	-	1
**α-haemolytic** ***Streptococcus***	35	10	11	9	5	-	16	3	3	2	2
***S. mitis***	3	-	1	1	1	-	-	-	-	-	3
***S. parasanguinis***	1	-	-	1	-	-	-	-	-	-	1
***S. pneumoniae***	1	1	-	-	-	-	1	-	-	-	-
***S. salivarius***	1	-	-	1	-	-	-	-	1	-	-
***V. parvula***	1	-	1	-	-	-	-	-	-	1	-
***Sum***	**118**	**30**	**21**	**28**	**21**	**16**	**28**	**5**	**7**	**16**	**35**

## Data Availability

Microbiome sequencing data have been submitted to the NCBI BioProject database under the accession number PRJNA1276981 (https://www.ncbi.nlm.nih.gov/bioproject/PRJNA1276981, accessed on 26 September 2025).

## Supplementary Materials

The following supporting information can be downloaded at https://www.mdpi.com/article/10.3390/ijms26209841/s1: References [62,63,64,65,66,67,68,69,70,71] are cited in the supplementary materials.

## Abbreviations

The following abbreviations are used in this manuscript:

BALF	Bronchoalveolar lavage fluid
IQR	Interquartile range
LRTI	Lower respiratory tract infection
NGS	Next-generation sequencing
ONT	Oxford Nanopore Technologies
OTU	Operational taxonomic unit
