# Supplementary material for: Assessment of Microbiome-Based Pathogen Detection Using Illumina Short-Read and Nanopore Long-Read Sequencing in 144 Patients Undergoing Bronchoalveolar Lavage in a University Hospital in Germany
Source: Int J Mol Sci. 2025 Oct 10;26(20):9841. doi: 10.3390/ijms26209841 (PMC12564420; doi:10.3390/ijms26209841)
Supplement: Supplementary file 1 [file ijms-26-09841-s001.zip › ijms-3754464-supplementary.pdf]

Supplemental material

Microbiome-Based Clinical Pathogen Detection in Bronchoalveolar Lavage Fluid Using Illumina Short-Read and Nanopore Long-Read Sequencing

Merle Bitter[1]*, Markus Weigel[1,2]*, Jan Philipp Mengel[1], Benjamin Ott[1], Anita Windhorst[3], Khodr Tello[4], Can Imirzalioglu[1,2,] and Torsten Hain[1,2,]****

[1] Institute of Medical Microbiology, Medical Microbiome-Metagenome Unit (M3U), Justus Liebig University Giessen, 35392 Giessen, Germany
[2] German Center for Infection Research (DZIF), Partner Site Giessen-Marburg-Langen, Justus Liebig University Giessen, 35392 Giessen, Germany
[3] Institute of Medical Informatics, Justus Liebig University Giessen, 35392 Giessen, Germany
[4] Department of Internal Medicine, Justus Liebig University Giessen, Universities of Giessen and Marburg Lung Center (UGMLC), German Center for Lung Research (DZL), 35392 Giessen, Germany

* Contributed equally
** Correspondence: CI: can.imirzalioglu@mikrobio.med.uni-giessen.de; TH: torsten.hain@mikrobio.med.uni-giessen.de

Content:

- Supplementary Table S1
- Supplementary Table S2
- Supplementary Table S3
- Supplementary Table S4
- Supplementary Figure S1
- Supplementary Figure S2
- Supplementary Figure S3
- Supplementary Figure S4
- Supplementary Figure S5

Supplementary Table S1: Bacterial species identified within this study which were classified as potential pathogen. A reference for the classification as potential pathogen is given in the right column.

Potential Pathogen	Reference
Bordetella bronchiseptica	Gujju et al., 2021 [62]
Citrobacter koseri	Yao et al., 2021 [63]
Escherichia coli	Dalhoff et al., 2017 [64]; Ewig et al., 2021 [65]
Haemophilus influenzae	Dalhoff et al., 2017 [64]; Ewig et al., 2021 [65]
Listeria monocytogenes	Koufakis et al., 2015 [66]
Prevotella oris	Viswanath et al., 2022 [67]
Pseudomonas aeruginosa	Dalhoff et al., 2017 [64]; Ewig et al., 2021 [65]
Ralstonia pickettii	Ryan et al., 2011 [68]
Serratia marcescens	Dalhoff et al., 2017 [64]
Staphylococcus aureus	Dalhoff et al., 2017 [64]; Ewig et al., 2021 [65]
Stenotrophomonas sp. MYb57	-
Stenotrophomonas maltophilia	Dalhoff et al., 2017 [64]
Streptococcus agalactiae	Furuta et al., 2022 [69]
Streptococcus pneumoniae	Dalhoff et al., 2017 [64]; Ewig et al., 2021 [65]
Streptococcus pseudopneumoniae	Garriss et al., 2019 [70]
Tropheryma whipplei	Shi et al., 2024 [71]

Supplementary Table S2. Summary of identified bacteria in culture-based diagnostics and the top three detections with a relative abundance above 3% for NGS sequencing. ONT sequenced samples had the additional constraint of a minimum of 10 sequence reads. Pathogenic bacteria a denoted in bold.

Sample	Culture-based Routine Diagnostics (24h to 48h)	Illumina Short-Read Sequencing (32h)	Nanopore Long-Read Sequencing (8h)
P002	***C. koseri*** (plenty)<br>*S. epidermidis* (mass)	*Corynebacterium* (3.1%)	***C. koseri*** (85.7%)
P003	None	None	None
P004	***S. aureus*** (plenty)<br>*S. mitis* (sparse)	*Staphylococcus* (76.1%)<br>*Streptococcus* (4.5%)	***S. aureus*** (85.0%)
P007	*E. faecium* (sparse)<br>Coagulase-negative *Staphylococcus* (sparse)	*Veillonella* (12.1%)	None
P008	None	None	Excluded
P009	Pharyngeal flora (sparse)	*Streptococcus* (39.8%)<br>*Veillonella* (3.3%)	None
P011	α-hemolytic *Streptococcus* (few)	*Gemella* (11.6%)<br>*Streptococcus* (6.4%)<br>*Rothia* (6.1%)	None
P013	***S. aureus*** (mass)	*Haemophilus* (4.5%)	***R. pickettii*** (7.4%)<br>*L. curvatus* (6.9%)<br>*S. hominis* (5.9%)
P014	***S. aureus*** (mass)	None	*P. myrsinacearum* (10.5%)
P105	α-hemolytic *Streptococcus* (plenty)<br>Saproph. *Neisseria* (few)	*Streptococcus* (21.6%)<br>*Veillonella* (16.1%)<br>*Prevotella* 7 (10.7%)	*V. atypica* (16.8%)<br>*S. mitis* (16.5%)<br>*S. salivarius* (12.1%)
P017	***S. pneumoniae*** (mass)	*Streptococcus* (99.9%)	***S. pneumoniae*** (100.0%)
P018	α-hemolytic *Streptococcus* (sparse)	*Streptococcus* (36.9%)<br>*Rothia* (18.3%)<br>*Enterococcus* (15.0%)	Excluded
P019	None	*Streptococcus* (13.6%)<br>*Absconditabacteriales* (7.9%)<br>*Alloprevotella* (7.4%)	Excluded
P021	None	*Veillonella* (7.6%)<br>*Prevotella* 7 (4.9%)<br>*Corynebacterium* (4.4%)	Excluded
P022	α-hemolytic *Streptococcus* (plenty)<br>Saproph. *Neisseria* (plenty)<br>***P. aeruginosa*** (sparse)	*Veillonella* (15.8%)<br>*Streptococcus* (10.1 %)<br>*Prevotella* 7 (7.9%)	*C. concisus* (17.6%)<br>*V. dispar* (13.9%)<br>*S. parasanguinis* (10.1%)
P023	α-hemolytic *Streptococcus* (plenty)<br>*R. mucilaginosa* (plenty)	*Streptococcus* (43.2%)<br>*Gemella* (33.6%)<br>*Rothia* (11.6%)	Excluded
P024	α-hemolytic *Streptococcus* (sparse)<br>***H. influenzae*** (mass)	*Haemophilus* (95.4%)	Excluded
P025	None	*Corynebacterium* (5.0%)	***S. aureus*** (93.0%)
P027	***S. aureus*** (mass)	*Staphylococcus* (53.3%)<br>*Prevotellaceae* (38.0%)<br>*Prevotella* 7 (7.3%)	Excluded
P028	α-hemolytic *Streptococcus* (plenty)<br>Saproph. *Neisseria* (plenty)	*Selenomonas* (8.0%)<br>*Alloprevotella* (7.6%)	Excluded

		Prevotella (7.5%)	
P029	α-hemolytic *Streptococcus* (few)	*Streptococcus* (12.6%)<br>*Porphyromonas* (3.6%)	*S. mitis* (10.3%)<br>*H. parainfluenzae* (9.0%)<br>*Selenomonas* sp. (8.8%)
P033	None	None	*L. sacchari* (37.5%)
P034	None	*Neisseria* (27.6%)<br>*Haemophilus* (18.1%)<br>*Streptococcus* (13.4%)	Excluded
P036	*Rothia* sp. (sparse)	*Rothia* (20.1%)	***H. influenzae*** (33.1%)<br>*H. parainfluenzae* (19.8%)<br>*N. macacae* (8.2.%)
P038	None	*Burkholderiales* (20.8%)<br>*Parvimonas* (12.6%)<br>*Neisseria* (5.7%)	Excluded
P039	*S. epidermidis* (sparse)	*Staphylococcus* (5.9%)<br>*Prevotella 7* (3.4%)	None
P040	α-hemolytic *Streptococcus* (plenty)<br>Saproph. *Neisseria* (plenty)	*Streptococcus* (10.3%)<br>*Alloprevotella* (9.7%)<br>*Prevotella* (9.6%)	Excluded
P041	α-hemolytic *Streptococcus* (plenty)<br>Saproph. *Neisseria* (plenty)	*Prevotella 7* (9.1%)<br>*Rothia* (8.9%)<br>*Streptococcus* (8.5%)	None
P042	None	None	*Streptococcus* sp. (12.3%)<br>*Selenomonas* sp. (10.0%)<br>*Streptococcus* sp. (9.1%)
P043	***P. aeruginosa*** (few)	*Pseudomonas* (40.4%)<br>*Enterobacteriaceae* (25.4%)	*S. mitis* (9.6%)<br>*G. sanguinis* (9.3%)<br>*V. atypica* (9.3%)
P044	***P. aeruginosa*** (plenty)	*Pseudomonas* (81.6%)	Excluded
P045	α-hemolytic *Streptococcus* (mass)<br>*Rothia* sp. (few	*Prevotella 7* (26.9%)<br>*Streptococcus* (12.9%)<br>*Veillonella* (8.1%)	Excluded
P048	*E. faecium* (few)	*Enterococcus* (62.0%)<br>*Rothia* (20.4%)<br>*Lacticaseibacillus* (3.5%)	Excluded
P049	*E. faecium* (few)	*Enterococcus* (83.6%)	None
P050	None	*Porphyromonas* (15.4%)<br>*Lactobacillales* (8.1%)<br>*Actinomyces* (4.3%)	Not Determined
P051	α-hemolytic *Streptococcus* (few)<br>*Rothia* sp. (sparse)<br>Coagulase-negative *Staphylococcus* (sparse)<br>*P. melaninogenica* (sparse)	*Veillonella* (26.1%)<br>*Streptococcus* (23.9%)<br>*Prevotella* 7 (8.6%)	*E. faecium* (90.0%)
P052	***S. aureus*** (few)	*Staphylococcus* (91.4%)	Excluded
P055	None	*Enterococcus* (4.6%)<br>*Rothia* (4.1%)	None
P057	None	*Selenomonas* (4.6%)<br>*Lactobacillales* (4.5%)<br>*Intrasporangiaceae* (3.2%)	Excluded
P058	Coagulase-negative *Staphylococcus* (sparse)	*Staphylococcus* (76.9%)<br>*Veillonella* (3.4%)	*S. epidermidis* (80.3%)

P059	None	*Streptococcus* (9.8%)<br>*Selenomonadaceae* (6.0%)<br>*Chryseobacterium* (3.6%)	None
P060	*S. epidermidis* (mass)	*Rothia* (34.7%)<br>*Streptococcus* (27.1%)<br>*Enterococcus* (14.6%)	*S. mitis* (42.8%)<br>*S. epidermidis* (21.4%)<br>*E. faecium* (20.6%)
P065	None	*Brevibacterium* (6.0%)<br>*Pseudomonas* (4.8%)<br>*Actinomyces* (3.3%)	Excluded
P070	α-hemolytic *Streptococcus* (sparse)	*Streptococcus* (47.3%)<br>*Rothia* (9.0 %)<br>*Limosilactobacillus* (6.7%)	***S. pseudopneumoniae*** (35.6%)<br>*Streptococcus* sp. (21.0%)<br>***S. agalactiae*** (18.4%)
P071	None	*Staphylococcus* (13.9%)<br>*Enterococcus* (8.5%)<br>*Corynebacterium* (6.6%)	Excluded
P072	***S. maltophilia*** (plenty)	*Stenotrophomonas* (95.4%)	***S. maltophilia*** (98.8%)
P073	None	*Staphylococcus* (6.9%)	Excluded
P074	α-hemolytic *Streptococcus* (sparse)	*Alloprevotella* (9.6%)<br>*Actinomyces* (7.3%)<br>*Gemella* (5.6%)	Not Determined
P075	α-hemolytic *Streptococcus* (sparse)<br>Coagulase-negative *Staphylococcus* (sparse)	*Streptococcus* (19.1%)<br>*Veillonella* (17.6%)<br>*Prevotella 7* (17.2%)	*V. atypica* (24.3%)<br>*S. mitis* (17.1%)<br>*L. gingivalis* (15.1%)
P076	α-hemolytic Streptococcus (few)	*Prevotella* (29.3%)<br>*Mycoplasma* (13.6%)<br>*Prevotellaceae* (13.0%)	*P. salivae* (21.6%)<br>***P. oris*** (19.2%)<br>*P. stomatis* (7.2%)
P077	None	*Streptococcus* (8.1%)<br>*Enterococcus* (5.9%)<br>*Haemophilus* (3.4%)	Excluded
P079	α-hemolytic *Streptococcus* (sparse)<br>*N. flava* (sparse)<br>*V. parvula* (sparse)<br>*S. parasanguinis* (sparse)	*Neisseria* (16.3%)<br>*Haemophilus* (11.6%)<br>*Veillonella* (10.3%)	*Streptococcus* sp. (15.4%)<br>*N. perflava* (14.7%)<br>*P. nanceiensis* (11.0 %)
P080	α-hemolytic *Streptococcus* (few)<br>*R. mucilaginosa* (few)	*Streptococcus* (25.8%)<br>*Veillonella* (16.5%)<br>*Prevotella 7* (12.0%)	***S. pseudopneumoniae*** (22.8%)<br>*S. mitis* (22.4%)<br>*V. dispar* (10.4%)
P082	α-hemolytic *Streptococcus* (mass)<br>Saproph. *Neisseria* (mass)<br>***E. coli*** (plenty)	*Prevotella 7* (24.4%)<br>*Veillonella* (22.6%)<br>*Streptococcus* (18.7%)	*Streptococcus* sp. (12.1%)<br>*S. oralis* (12.1%)<br>*V. dispar* (10.5%)
P083	α-hemolytic *Streptococcus* (few)<br>Saproph. *Neisseria* (sparse)<br>Coagulase-negative *Staphylococcus* (sparse)	*Prevotella 7* (17.3%)<br>*Veillonella* (12.4%)<br>*Actinomyces* (11.6%)	Not Determined
P085	None	*Staphylococcus* (7.4%)<br>*Enterobacteriaceae* (6.4%)	*M. osloensis* (63,83%)
P086	None	*Streptococcus* (13.7%)<br>*Pseudomonas* (5.3%)	None
P088	*S. epidermidis* (sparse)<br>*A. odontolyticus* (sparse)<br>*L. reuteri* (sparse)	*Lactobacillus* (12.4%)<br>*Veillonella* (7.9%)	Excluded
P089	*S. mitis* (sparse)	*Rothia* (15.1%)<br>*Veillonella* (11.4%)	None

		Prevotellaceae (5.9%)	
P091	*S. mitis* (sparse)<br>***S. aureus*** (sparse)<br>*R. mucilaginosa* (few)	*Rothia* (21.3%)<br>*Streptococcus* (16.2%)<br>*Prevotella 7* (16.1 %)	*Streptococcus* sp. (21.6%)<br>*S. peroris* (17.8%)<br>*V. atypica* (16.8%)
P092	None	*Prevotella* 7 (15.6%)<br>*Corynebacterium* (13.1%)<br>*Rothia* (5.3%)	None
P093	α-hemolytic *Streptococcus* (mass)<br>*N. flava* (mass)<br>***S. aureus*** (sparse)<br>*P. melaninogenica* (plenty)	*Neisseria* (35.9%)<br>*Streptococcus* (22.5%)<br>*Haemophilus* (7.8%)	*N. flavescens* (32.3%)<br>***S. pneumoniae*** (27.3%)<br>*H. haemolyticus* (7.3%)
P094	Pharyngeal flora (few)	*Micrococcales* (18.5%)<br>*Prevotella 7* (16.2%)<br>*Veillonella* (12.6%)	***T. whipplei*** (12.8%)<br>*V. dispar* (12.1%)<br>*V. atypica* (9.9%)
P095	***P. aeruginosa*** (mass)	*Pseudomonas* (77.1%)<br>*Stenotrophomonas* (8.1%)<br>*Neisseriaceae* (7.6%)	***P. aeruginosa*** (93.1%)<br>***S. maltophilia*** (4.5%)
P096	None	*Corynebacterium* (47.7%)<br>*Enterococcus* (23.6%)<br>*Neisseriaceae* (10.5%)	*E. faecium* (35.0%)<br>*S. epidermidis* (18.5%)<br>*C. tuberculostearicum* (8.3%)
P097	α-hemolytic *Streptococcus* (few)<br>Coagulase-negative *Staphylococcus* (few)<br>***B. bronchiseptica*** (few)	*Micrococcales* (93.4%)<br>*Bordetella* (4.4%)	***T. whipplei*** (92.7%)<br>***B. bronchiseptica*** (2.9%)
P098	None	*Enterococcus* (5.1%)<br>*Acinetobacter* (3.5%)<br>*Alphaproteobacteria* (3.3%)	None
P100	α-hemolytic *Streptococcus* (sparse)<br>Saproph. *Neisseria* (sparse)	*Paracoccus* (26.0%)<br>*Alloprevotella* (12.1%)<br>*Neisseria* (9.6%)	*P. contaminans* (43.0%)<br>*N. perflava* (16.7 %)<br>*V. atypica* (4.4%)
P101	*E. faecium*	*Enterococcus* (5.6%)<br>*Pediococcus* (3.1%)	*M. osloensis* (10.4%)<br>*E. faecium* (7.8%)
P102	α-hemolytic *Streptococcus* (mass)<br>***S. aureus*** (few)<br>*P. melaninogenica* (few)	*Rothia* (11.6%)<br>*Fusobacterium* (11.1%)<br>*Bacteroidales* (10.9%)	Not Determined
P103	None	*Veillonella* (16.6%)<br>*Prevotella 7* (6.1%)	*V. dispar* (5.8%)<br>*V. parvula* (5.3%)<br>*M. osloensis* (4.6%)
P104	α-hemolytic *Streptococcus* (plenty)	*Prevotella 7* (19.2%)<br>*Streptococcus* (14.9%)<br>*Rothia* (14.0%)	*Streptococcus* sp. (18.0%)<br>*S. mitis* (13.4%)<br>*S. oralis* (9.5%)
P105	None	*Streptococcus* (3.2%)<br>*Haemophilus* (3.0%)	*A. segnis* (10.7%)<br>*S. oralis* (3.8%)<br>*F. nucleatum* (3.1%)
P106	None	*Fusobacterium* (18.1%)<br>*Limosilactobacillus* (9.2%)<br>*Parvimonas* (8.1%)	*P. micra* (16.8%)<br>*S. mitis* (7.0%)<br>*C. rectus* (4.7%)
P107	***P. aeruginosa*** (plenty)	*Leptotrichia* (9.1%)<br>*Veillonella* (6.1%)<br>*Prevotellaceae* (5.0%)	*H. haemolyticus* (6.0%)<br>*S. mitis* (4.2%)<br>*Leptotrichia* sp. 212 (3.6%)
P108	None	None	*M. osloensis* (8.2%)
P109	None	*Veillonella* (12.4%)<br>*Streptococcus* (8.7%)	*L. lactis* (48.5%)<br>*S. saccharolyticus* (10.8 %)

		Rothia (7.1%)	*R. inusitata* (6.6%)
P110	***S. maltophilia*** (sparse)	*Stenotrophomonas* (37.5%)<br>*Corynebacterium* (23.3%)<br>*Streptococcus* (20.4%)	***Stenotrophomonas* sp. *MYb57*** (56.5%)<br>*S. mitis* (34.3%)
P111	None	*Porphyromonas* (42.6%)<br>*Veillonella* (11.5%)<br>*Alloprevotella* (8.5%)	*P. stomatis* (23.3%)<br>*V. atypica* (15.2%)<br>*P. pasteri* (10.1%)
P112	*R. mucilaginosa* (plenty)<br>α-hemolytic *Streptococcus* (sparse)	*Streptococcus* (20.1%)<br>*Veillonella* (19.8%)<br>*Rothia* (13.2%)	*Streptococcus* sp. (43.9%)<br>*V. dispar* (27.7%)<br>*C. concisus* (8.1%)
P113	None	*Streptococcus* (17.6%)<br>*Prevotella 7* (8.9%)<br>*Veillonella* (7.8%)	*Streptococcus* sp. (13.1%)<br>*S. oralis* (9.8%)<br>*G. elegans* (9.1%)
P114	None	*Streptococcus* (24.9%)<br>*Fusobacterium* (17.5%)<br>*Gemella* (13.2%)	*S. anginosus* (34.1%)<br>*G. morbillorum* (18.0%)<br>*P. micra* (13.4%)
P115	***E. coli*** (plenty)<br>*S. salivarius* (plenty)<br>*P. melaninogenica* (few)	*Veillonella* (15.1%)<br>*Prevotella 7* (12.4%)<br>*Streptococcus* (8.9%)	*S. oralis* (12.5%)<br>*V. atypica* (12.0%)<br>*V. dispar* (12.0%)
P116	*E. faecalis* (sparse)	*Streptococcus* (19.9%)<br>*Veillonella* (6.2%)<br>*Haemophilus* (3.2%)	***L. monocytogenes*** (11.0%)<br>*T. bourrellyi* (6.9%)
P117	None	*Corynebacterium* (6.6%)	*B. deliciosa* (5.6%)
P118	None	None	*B. subtilis* (6.8%)<br>*O. sancta* (3.8%)
P119	α-hemolytic *Streptococcus* (sparse)	*Streptococcus* (12.5%)<br>*Rothia* (8.8%)<br>*Haemophilus* (8.2%)	*H. parainfluenzae* (30.8%)<br>*S. infantis* (4.7%)<br>*S. sanguinis* (4.4%)
P120	α-hemolytic *Streptococcus* (plenty)<br>Saproph. *Neisseria* (plenty)<br>*R. mucilaginosa* (plenty)	*Prevotella 7* (27.9%)<br>*Staphylococcus* (26.2%)<br>*Rothia* (16.9%)	*S. epidermidis* (37.7%)<br>*S. mitis* (16.9%)<br>*S. oralis* (16.3%)
P123	Coagulase-negative *Staphylococcus* (few)	*Prevotella 7* (10.6 %)<br>*Saccharimonadaceae* (10.6 %)<br>*Veillonella* (8.9%)	*Streptococcus* sp. (11.7%)<br>*M. micronuciformis* (8.8%)<br>*S. salivarius* (8.3%)
P124	Coagulase-negative *Staphylococcus* (plenty)<br>*E. faecalis* (few)	*Staphylococcus* (38.2%)<br>*Enterococcus* (22.7%)<br>*Corynebacterium* (8.6%)	*S. epidermidis* (46.8%)<br>*E. faecalis* (29.4%)<br>***L. monocytogenes*** (3.3%)
P125	None	*Streptococcus* (37.1%)<br>*Rothia* (14.2%)<br>*Veillonella* (9.3%)	*S. mitis* (38.0%)<br>*H. parainfluenzae* (28.4%)<br>*V. atypica* (6.2%)
P126	α-hemolytic *Streptococcus* (mass)<br>***S. aureus*** (mass)<br>***S. marcescens*** (plenty)<br>*H. parahaemolyticus* (mass)	*Haemophilus* (20.6%)<br>*Prevotella 7* (15.6%)<br>*Streptococcus* (12.7%)	*H. parahaemolyticus* (46.3%)<br>*S. mitis* (6.7%)<br>*H. parainfluenzae* (5.3%)
P127	α-hemolytic *Streptococcus* (sparse)	*Prevotella 7* (17.7%)<br>*Veillonella* (7.9%)<br>*Staphylococcus* (6.5%)	*Streptococcus* sp. (22.3%)<br>*V. dispar* (11.5%)<br>*V. atypica* (10.0%)
P128	α-hemolytic *Streptococcus* (plenty)<br>Saproph. *Neisseria* (plenty)<br>Coagulase-negative *Staphylococcus* (sparse)	*Streptococcus* (24.1%)<br>*Rothia* (19.5%)<br>*Prevotella 7* (8.8%)	*S. mitis* (36.8%)<br>*S. infantis* (11.3%)<br>*S. oralis* (10.0%)

P130	None	*Streptococcus* (4.2%)<br>*Prevotella 7* (3.9%)<br>*Pirellulaceae* (3.9%)	None
P131	None	*Acetobacteraceae* (5.6%)<br>*Brachybacterium* (4.0%)<br>*Staphylococcus* (3.8%)	None
P133	***S. maltophilia*** (mass)<br>α-hemolytic *Streptococcus* (plenty)	*Stenotrophomonas* (58.0%)<br>*Prevotella 7* (14.2.%)<br>*Streptococcus* (10.0%)	***S. maltophilia*** (91.9%)
P134	None	None	None
P135	None	*Rothia* (6.9%)<br>*Haemophilus* (6.1%)<br>*Streptococcus* (4.9%)	None
P137	None	*Veillonella* (37.2%)<br>*Prevotella* (7.9%)<br>*Neisseriaceae* (7.2%)	*V. parvula* (48.0%)<br>*V. rogosae* (11.0%)<br>*N. elongata* (4.6%)
P138	α-hemolytic *Streptococcus* (plenty)<br>Saproph. *Neisseria* (plenty)	*Micrococcales* (27.0%)<br>*Veillonella* (15.9%)<br>*Prevotella* 7 (13.4%)	*V. atypica* (22.8%)<br>***T. whipplei*** (14.9%)<br>*M. micronuciformis* (12.9%)
P140	***C. koseri*** (plenty)	*Veillonella* (23.2%)	***C. koseri*** (87.1%)<br>*V. dispar* (4.3%)
P141	α-hemolytic *Streptococcus* (few)<br>Saproph. *Neisseria* (few)	*Veillonella* (16.9 %)<br>*Prevotella 7* (12.9%)<br>*Streptococcus* (11.9%)	*V. atypica* (17.0%)<br>*S. salivarius* (12.7%)<br>*V. dispar* (12.1%)
P142	None	*Staphylococcus* (4.9%)<br>*Pseudomonas* (3.4%)	*T. erythraeum* (32,02%)
P143	***S. aureus*** (mass)	*Streptococcus* (22.9%)<br>*Staphylococcus* (16.1%)<br>*Prevotella 7* (14.1%)	***S. pneumoniae*** (57.2%)<br>***S. aureus*** (9.2%)<br>*S. mitis* (3.0%)
P144	***S. aureus*** (few)<br>α-hemolytic *Streptococcus* (few)<br>*P. melaninogenica* (few)<br>*L. salivarius* (sparse)	*Streptococcus* (34.4%)<br>*Rothia* (24.4%)<br>*Prevotella 7* (9.2%)	*S. mitis* (55.3%)<br>***S. pseudopneumoniae*** (16.9%)<br>*G. haemolysans* (6.6%)

Supplementary Table S3. Overview of the number of BALF samples classified by their composition for Illumina short-read and ONT long-read sequencing.

	**Negative Culture**		**Commensals in Culture**		**Potential Pathogens in Culture**	
	**Illumina**	**ONT**	**Illumina**	**ONT**	**Illumina**	**ONT**
**Monomicrobial**	0	4	3	8	10	13
**Polymicrobial**	6	9	17	14	7	5
**Multimicrobia**	20	9	19	7	6	2
**Low-Biomass**	13	6	2	3	3	1

Supplementary Table S4. OTUs removed as contamination because of their association with the negative controls used during Illumina short-read sequencing.

OTU ID	Genus
OTU0001	*Escherichia-Shigella*
OTU0007	*Pseudomonas*
OTU0008	*Pseudomonas*
OTU0010	*Enhydrobacter*
OTU0012	*Cutibacterium*
OTU0019	*Rhodobacteraceae* unclassified
OTU0042	*Micrococcus*
OTU0053	*Corynebacterium*
OTU0054	*Micrococcales* unclassified
OTU0069	*Methylobacterium-Methylorubrum*
OTU0077	*Brevundimonas*
OTU0079	*Corynebacteriales* unclassified
OTU0093	*Acinetobacter*
OTU0097	*Xanthobacteraceae* unclassified
OTU0098	*Kocuria*
OTU0107	*Cupriavidus*
OTU0108	*Methylobacterium-Methylorubrum*
OTU0109	*Lactococcus*
OTU0122	*Comamonadaceae* unclassified
OTU0125	*Pontibacter*
OTU0143	*Propionibacteriaceae* unclassified
OTU0200	*Allorhizobium-Neorhizobium-Pararhizobium-Rhizobium*
OTU0209	*Chryseobacterium*
OTU0210	*Sphingomonas*
OTU0212	*Chryseobacterium*
OTU0213	*Acidibacter*
OTU0254	*Burkholderia-Caballeronia-Paraburkholderia*
OTU0271	*Yersiniaceae* unclassified
OTU0290	*Sphingomonas*
OTU0463	*Bacteroides*
OTU0481	*Ruminococcus*
OTU0533	*Lachnospiraceae* unclassified

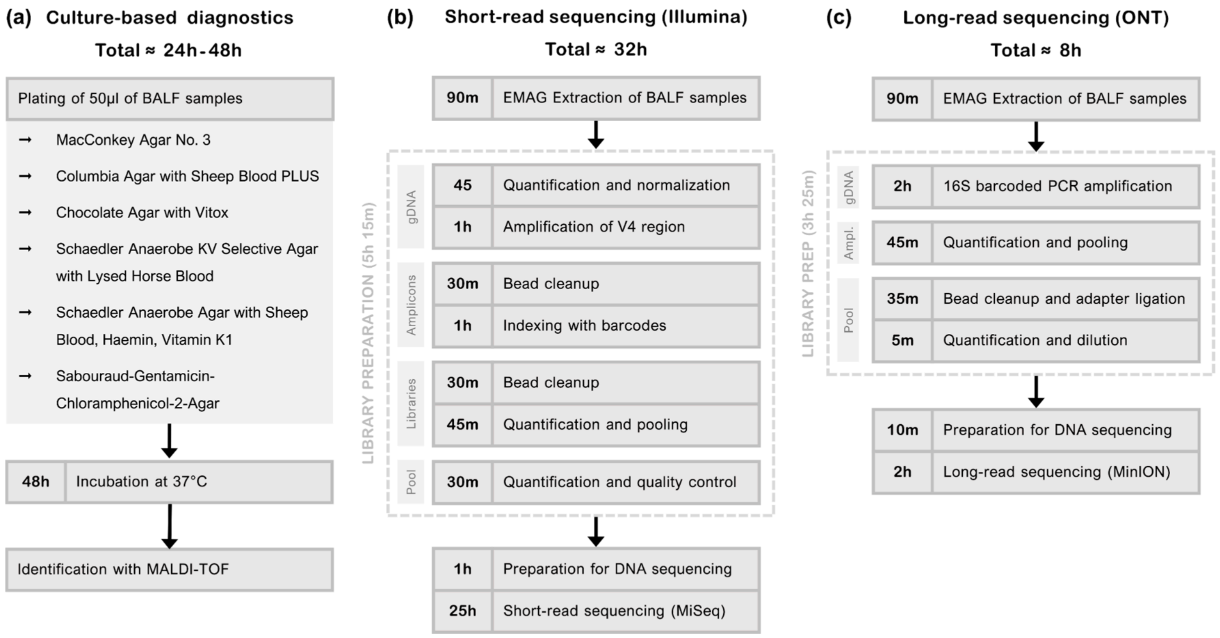

Supplementary Figure S1. Overview of the workflows and time estimated for completion of (a) culture-based diagnostics, (b) Illumina sequencing and (c) ONT sequencing.

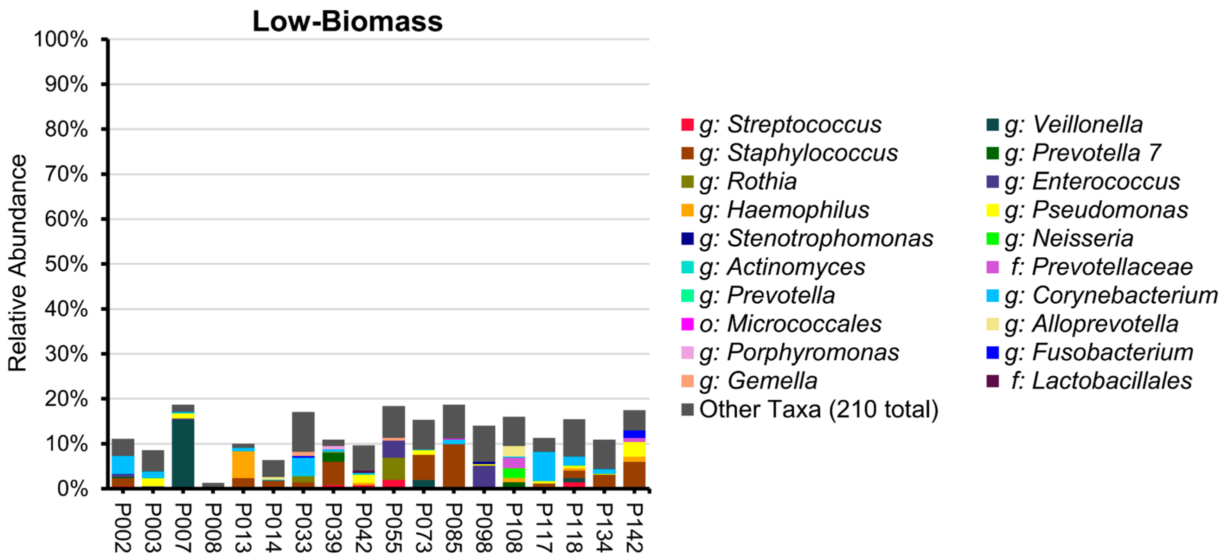

Supplementary Figure S2. Illumina short-read sequenced BALF samples classified as low-biomass.

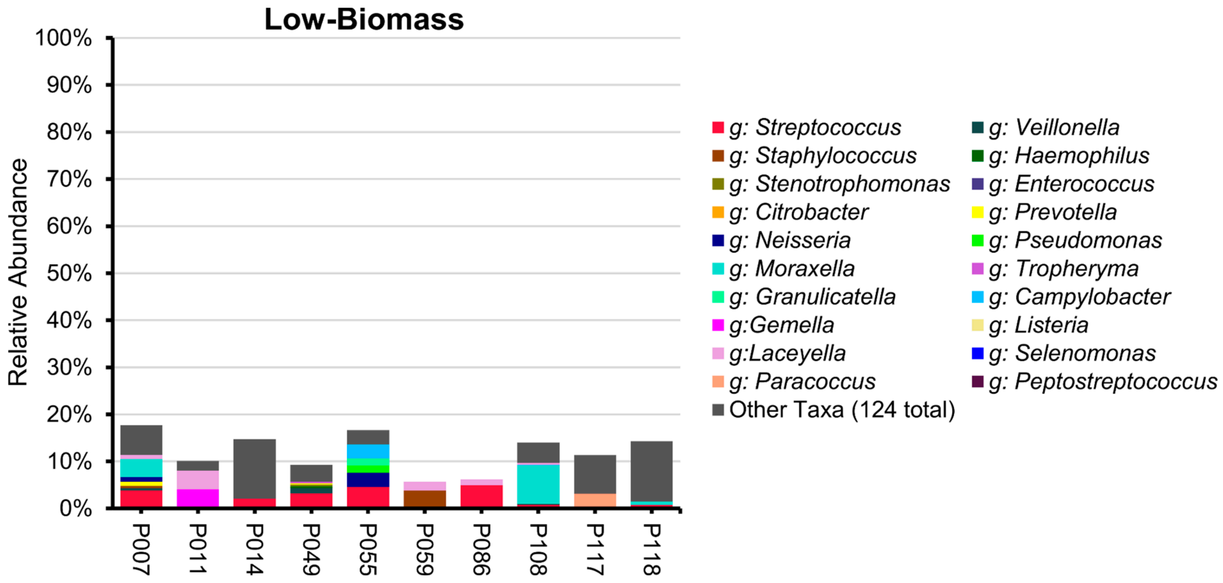

Supplementary Figure S3. ONT long-read sequenced BALF samples classified as low-biomass.

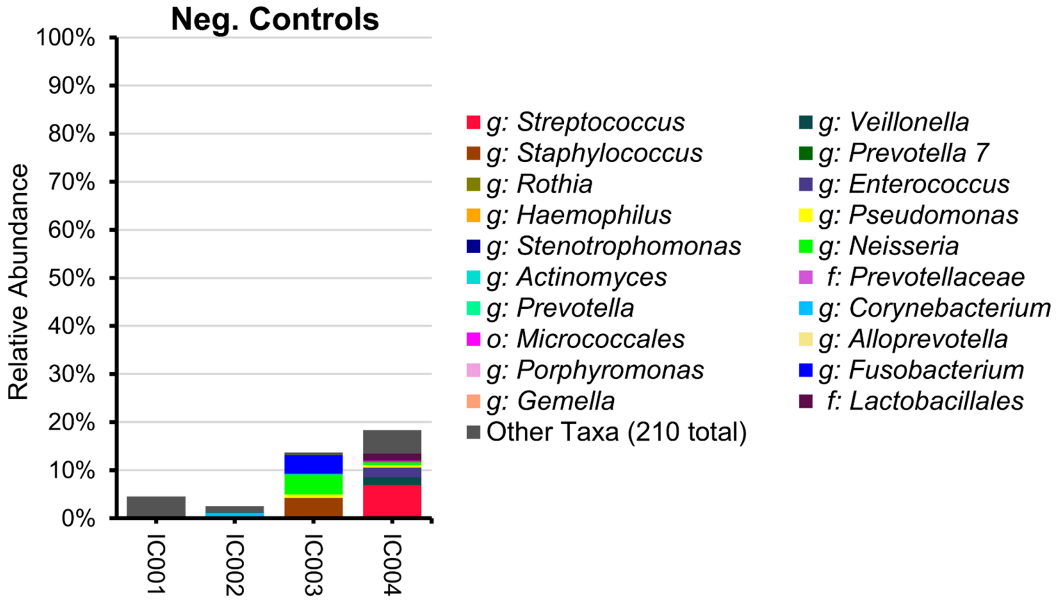

Supplementary Figure S4. Remaining negative controls for Illumina short-read sequencing after removal of detected contaminations (Supplementary Table S3).

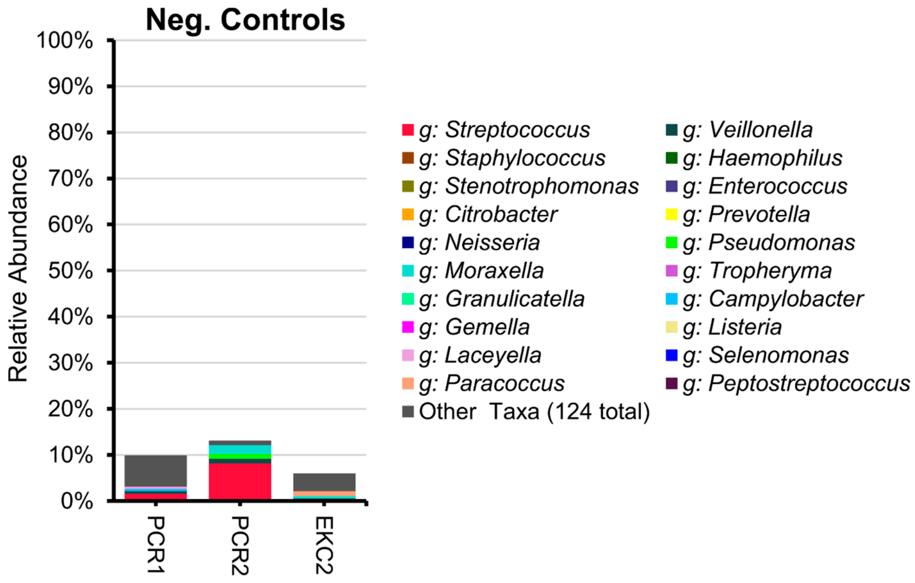

Supplementary Figure S5. Remaining negative controls for ONT long-read sequencing after removal of contaminations by *Delftia acidovorans*, *Escherichia coli*, *Paracoccus angustae* and *Paracoccus marinus*.
